# Exosomes containing long non-coding RNA AGAP2-AS1 promote the differentiation of CD4^+^ T cells through the miR-424-5p/SGK1 axis in psoriasis

**DOI:** 10.3389/fgene.2025.1521470

**Published:** 2025-05-30

**Authors:** Ziqi Yuan, Xue Zeng, Xiwei Zhang, Chenglai Xia, Xuebiao Peng

**Affiliations:** ^1^ Department of Dermatology, Nanfang Hospital, Southern Medical University, Guangzhou, China; ^2^ Foshan Maternity and Child Healthcare Hospital, Foshan, China

**Keywords:** long non-coding RNAs, micrornas, psoriasis, t cells, exosomes

## Abstract

**Background:**

Long non-coding RNAs (lncRNAs) have been implicated in the pathogenesis of autoimmune diseases. Our previous research demonstrated that AGAP2-AS1 in keratinocytes is involved in the pathogenesis of psoriasis, but its effect on CD4^+^ T cell differentiation remains unclear.

**Methods:**

Exosomes were extracted from HaCaT cells using a reagent kit and verified by TEM (Transmission Electron Microscope), NTA (Nanoparticle Tracking Analysis), and Western Blot. We incubated exosomes with CD4^+^ T cells and detected the distribution of AGAP2-AS1 by fluorescence microscopy. Flow cytometry and ELISA were used to detect CD4^+^ T cell differentiation. In addition, the relationship between AGAP2-AS1/miR-424-5p/SGK1 and its effect on CD4^+^ T cell differentiation were confirmed by bioinformatics analysis, dual luciferase reporter gene experiments, quantitative real-time PCR, flow cytometry, and ELISA.

**Results:**

We found that exosomes derived from TNF-α-treated HaCaTs were able to deliver AGAP2-AS1 to CD4^+^ T cells, promoting Th1 and Th17 differentiation. In CD4^+^ T cells, AGAP2-AS1 promotes Th1 and Th17 differentiation via the miR-424-5p/SGK1 axis.

**Conclusion:**

Psoriatic HaCaTs deliver AGAP2-AS1 to CD4^+^ T cells via exosomes, inducing Th1 and Th17 differentiation through the miR-424-5p/SGK1 axis, thereby promoting the progression of psoriasis. These findings provide novel insights into the pathogenesis of psoriasis and potential therapeutic targets.

## 1 Introduction

Psoriasis is a chronic inflammatory disease affecting approximately 3% of the global population (Armstrong and Read, 2020). Despite its prevalence, there is currently no cure for this condition. Patients with psoriasis suffer from immune system dysregulation, characterized by various differentiated subpopulations of CD4^+^ T cells. Psoriatic peripheral blood and skin lesions have been shown to contain higher proportions of different CD4^+^ T cells (including Th1, Th2, Th17, and Tfh) than healthy donors (Gao et al., 2023). The pathogenesis of psoriasis is primarily driven by Th1 and Th17 cells and their secretion of pro-inflammatory factors (Kim et al., 2022). Tregs are dysregulated in number and function in patients with psoriasis, resulting in reduced immunosuppression (Nussbaum et al., 2021). The persistence of a high expression of TRM markers (CD4, CD8, CD103, CD69, CD49, CXCR6) in the dermis may result in the rapid recurrence of lesions after discontinuation of topical treatment (Kasprowicz-Furmańczyk et al., 2022). It has been shown that a multilayered network of coding RNAs, as well as non-coding RNAs, may be involved in regulating the overall function of T cells (Pagani et al., 2013). Still, the mechanism by which lncRNAs affect CD4^+^ T cell differentiation remains incompletely explored.

In psoriasis, the epidermis undergoes excessive proliferation, leading to the formation of characteristic scaly erythematous plaques. Beyond its physical and chemical barrier function, the epidermis also exhibits significant immunological activity. In particular, epidermal cells are an active part of the immunomodulatory skin microenvironment, which can control the threshold of inflammatory processes in the skin by inhibiting T-cell proliferation and cytokine production (Seiringer et al., 2021). However, how epidermal cells interact with CD4^+^ T cells is still not fully explored.

Exosomes have emerged as important mediators of intercellular communication in various biological processes, including skin homeostasis and disease. These extracellular vesicles, ranging in size from 30 nm to 150nm, facilitate communication between skin cells and regulate the skin microenvironment (He et al., 2018). Exosomes can carry diverse cargo, including proteins, miRNAs, and lncRNAs, thereby transferring biological information between cells. The abnormal production, secretion, or content disorder of exosomes have been confirmed to mediate the formation of chronic inflammatory microenvironments., and they are also involved in regulating the pathogenesis of a variety of diseases such as psoriasis, systemic lupus erythematosus, and systemic sclerosis (Fang et al., 2023; Fei et al., 2022; Zhang et al., 2023). Keratinocyte-derived exosomes have multi-dimensional biological functions and are involved in wound repair (Cheng et al., 2008), matrix remodeling (Chavez-Muñoz et al., 2008), and the regulation of the immune system (Cai et al., 2017). IL-23 and TNF-α mRNA expression levels were increased in exosomes from psoriasis patients (Sawamura et al., 2023), and IL-17A levels were significantly upregulated in exosomes from moderately severe patients compared to mild patients (Cordonnier et al., 2019), suggesting that exosomes can activate the IL-23/IL-17A-Th17 axis in receptor cells by delivering inflammatory mediators. Exosome-mediated communication between activated keratinocytes and skin-infiltrating immune cells promotes the progression of psoriatic disease (Jiang et al., 2019), but whether or not lncRNA in epidermal cells affects CD4^+^ T cell differentiation based on the level of exosomes remains to be fully explored.

In our previous research, we demonstrated that AGAP2-AS1 is elevated in psoriatic lesions (Xian et al., 2022). Building on this finding, by modeling the internal environment of psoriasis, we hypothesize that TNF-α-treated HaCaTs-derived exosomes can deliver AGAP2-AS1 to CD4^+^ T cells, potentially promoting CD4^+^ T cell differentiation towards Th1 and Th17 phenotypes. We further propose that this process may be mediated via the AGAP2-AS1/miR-424-5p/SGK1 axis.

## 2 Materials and reagents

### 2.1 Cell culture and cell treatment

Human immortalized keratinocytes cell line HaCaT was a kind gift from Postdoc Niu Bo, Foshan Maternity and Child Healthcare Hospital, Foshan 528,000, China. HaCaT cells were kept in a DMEM medium supplemented with 10% FBS. When the cell density reached 70%–80%, TNF-α (100 μg/L) was added to the serum-free medium to simulate the inflammatory state. The cells and cell supernatants were then harvested for use in the next step of the experiment. CD4^+^ T cells were cultured with a T cell-specific medium (10,981, STEMCELL), and Human CD3/CD28 T cell activator (10,971, 25 μL/mL, STEMCELL) and recombinant human IL-2 (78,220, 10 ng/mL, STEMCELL) for activation and expansion. In the experimental group, additional interventions such as the addition of exosomes from different treatment groups or transfection with AGAP2-AS1, miR-424-5p, etc., were performed. After 5 days of incubation, the cells were collected for the next step of the experiment. HEK-293T cells were cultured in DMEM medium supplemented with 10% fetal bovine serum. All cells were cultured under conditions of 37°C and 5% CO_2_.

### 2.2 Patients’ cell isolation

This study was approved by the ethics committee of the Nanfang Hospital (NFEC-202302-K15). Informed consent for publication was obtained from all participants. We selected ten healthy volunteers between the ages of 18 and 50 who did not have autoimmune diseases and collected peripheral blood samples using a standardized diagnostic procedure. Human peripheral blood mononuclear cells (PBMCs) were isolated from the peripheral blood by Ficoll density gradient centrifugation. CD4^+^ T cells were isolated from PBMCs using Miltenyi beads following the manufacturer’s instructions (130-096-533, Miltenyi Biotec).

### 2.3 CCK-8 assay

The proliferation ability of HaCaT cells was detected by the CCK8 assay (GK10001, Dojindo Laboratories, Kumamoto, Japan). Cells were seeded in a 96-well plate (7 × 10^3^ cells/well). 24 h later, CCK-8 (10 μL/well) reagent was added to each well at the same time. The culture was incubated for 4 h at 37 °C away from light, and cell viability-related absorbance at 450 nm was measured with ultraviolet-visible spectrophotometry (BioTek, Winooski, VT, United States).

### 2.4 Isolation and characterization of exosome

HaCaT cells were cultured in an exosome-free DMEM medium with or without TNF-α for 24 h. Culture supernatants were collected for exosome isolation. Exosomes in the cell culture supernatant were isolated using an Exosome Extraction and Purification Kit according to the manufacturer’s specifications (UR52121, Umibio). Briefly, the culture supernatants were centrifuged at 3,000 × g for 10 min at 4°C, and then the supernatant was mixed with the exosome concentration solution at a volume ratio of 4:1 for 7 h. After centrifugation at 10,000 × g for 60 min at 4°C, the exosomes were isolated and resuspended in PBS. Subsequently, the morphological characteristics of the exosomes were then observed using an HT7700 transmission electron microscope (Hitachi, Japan). The expression levels of exosome-positive markers (CD63 and TSG101) were detected by Western blot analysis. Particle size and concentration of exosomes were measured by nanoparticle tracking analysis (NTA) using a ZetaView_Particle Metrix (Particle Metrix, PMX-120, Germany).

### 2.5 Exosome uptake assay

Exosomes were labeled with the fluorescent linker PKH67 (UR52303, Umibo) according to the manufacturer’s instructions. Briefly, 5 µL of PKH67 and 100 µg of exosomes were added to 45 µL of diluent C. After mixing uniformly, let stand at room temperature in the dark for 10 min. Excess dye was removed using ultrafiltration. The fluorescence-marked exosomes were then added to CD4^+^ T cells in 24-well plates (1 × 10^6^ cells/well) and incubated overnight. Non-labeled exosomes were used to control. The next day, the cells were washed and examined by fluorescent microscopy.

### 2.6 Flow cytometry

In the last 7 hours of co-incubation of CD4^+^ T cells and exosomes, cells were activated with a cell stimulation cocktail (E-CK-A091, Elabscience) at 1 × 10^6^ cells/mL in a 96-well plate and incubated for 0.5–1 h, then Protein Transport Inhibitor MIX (E-CK-A091, Elabscience) was added and incubated for 5–6 h. For cell membrane staining, after two washes with cell staining buffer (E-CK-A107, Elabscience), the suspended cells were stained with CD4 antibodies (17-0,049-42, eBioscience). For intracellular cytokine staining, cells were fixed with fixation buffer (E-CK-A109, Elabscience) and then stained intracellularly with antibodies against human IFN-γ (E-AB-F1196C, Elabscience) and IL-17A (E-AB-F1173D, Elabscience). After washing the cells twice, the ratio of Th1 and Th17 cell populations was analyzed using a FACSCalibur flow cytometer, while a fluorescently labeled isotype control was used to determine background fluorescence and to exclude non-specific binding.

### 2.7 Western blot

Exosomes were denatured by incubation with loading buffer for 30 min at room temperature, cells were lysed in RIPA lysis buffer (BIOTEK, Shanghai, China) for 30 min on ice, and total protein content was quantified using the BCA protein assay kit (Pierce, Rockford, IL). Denatured samples were resolved on 10% or 12% SDS-polyacrylamide gels and transferred onto Polyvinylidene Difluoride (PVDF) membranes (Millipore, MA, United States) using equipment from Bio-Rad (Hercules, CA, United States). After blocking with 5% skim milk in TBST buffer for 2 h at room temperature, the membranes were incubated with primary antibodies overnight at 4°C. The primary antibodies used include SGK1 (UpingBio Technology Co., Ltd.), CD63 (1:1,000, #52090), TSG101 (1:1,000, Ab125011), Calnexin (1:1,000, #2433), GAPDH (Cell Signaling Technology). The membranes were then incubated with the appropriate horseradish peroxidase-conjugated secondary antibodies for 2 h at room temperature. Finally, the signals were visualized using a chemiluminescence solution (Pierce, IL, United States and photographed using a Bio-Rad gel imaging system.

### 2.8 ELISA

The ELISA kits were used to measure IFN-γ (E-EL-H0108c) and IL-17A (E-EL-H5812c) in the culture supernatants according to the manufacturer’s instructions.

### 2.9 Cell transfection

Small interfering RNA (siRNA) targeting the AGAP2-AS1, SGK1 gene and non-targeting siRNA control, miR- 424–5p mimics, miR-424–5p inhibitor and matched negative control (NC mimics or NC inhibitor) were purchased from Guangzhou Shuangquan Biological Technology Co., Ltd. Transfection reagents were performed using CALNPTM RNAi *in vitro* (D-nano) according to the manufacturer’s instructions. Lentivirus carrying green fluorescent protein was used to transfect the lentivirus encoding AGAP2-AS1 (LV-AGAP2-AS1) and empty vector (LV-NC) into peripheral blood CD4^+^ T cells. Lentivirus, empty vector, and transfection reagent were purchased from Genechem Co. Ltd. (Shanghai, China).

### 2.10 RNA extraction and quantitative real-time

Total RNA was extracted by Total RNA isolation kit (Foregene China). PrimeScript ™ The RT reagent kit (RR036A, Takara, Japan) was used to reverse transcribe RNA into cDNA, and mRNA quantification was performed using the SYBR Premix Ex Taq Kit (RR820A, Takara, Japan) on the CFX96 touch q-PCR system (BIO-RAD, United States). Calculate the relative expression level using the 2^−△△Ct^ method, with normalization for GADPH (mRNA) or U6 (miRNA) expression. Specific primers are listed in Table 1. The primers for miRNA qRT-PCR were obtained from RiboBio Co. Ltd. (Guangzhou, China).

**TABLE 1:** Primers were used for quantitative real-time in this study.

Gene symbol	Forward primer (5′→3′)	Reverse primer (5′→3′)
AGAP2-AS1	TAC​CTT​GAC​CTT​GCT​GCT​CTC	TGT​CCC​TTA​ATG​ACC​CCA​TCC
SGK1	GCT​GAA​ATA​GCC​AGT​GCC​TTG​G	GTT​CTC​CTT​GCA​GAG​TCC​GAA​G
IFN-γ	TGA​CTT​GAA​TGT​CCA​ACG​CAA​AGC	CGA​CCT​CGA​AAC​AGC​ATC​TGA​CTC
IL-6	AGT​GAG​GAA​CAA​GCC​AGA​GC	ACT​CCT​TAA​AGC​TGC​GCA​GA
RORγt	AGA​TAC​CCT​CAC​CTA​CAC​CTT​G	CCG​CTC​AGG​GCT​GTA​TTC​AA
T-bet	GCC​AAA​GGA​TTC​CGG​GAG​AA	CCT​GGG​GAA​CCA​CAT​CCT​TC
Ccl20	TGC​TGT​ACC​AAG​AGT​TTG​CTC	CGC​ACA​CAG​ACA​ACT​TTT​TCT​TT
IL-1β	TGA​TGG​CTT​ATT​ACA​GTG​GCA​A	GTC​CAT​GGC​CAC​AAC​AAC​TG
GADPH	CTC​CTC​CTG​TTC​GAC​AGT​CAG​C	CCC​AAT​ACG​ACC​AAA​TCC​GTT

### 2.11 Dual-luciferase reporter assay

The AGAP2-AS1 or SGK1 sequence, containing the predicted miR-424-5p binding site for the mutant (MUT) or wild type (WT), was synthesized and cloned into the psiCHECK-2 luciferase reporter vector (Shuangquan Biotechnology Co., Ltd., Guangzhou, China). HEK-293 T cells were inoculated into a 96-well plate at a density of 5 × 10^3^ cells/well and incubated overnight. Subsequently, the recombinant construct was transfected into the HEK-293 T cells along with miR-424-5p or NC mimics using Lipofectamine 3,000 (Invitrogen, United States). After 48 h of transfection, the relative activity of luciferase was measured using a dual-luciferase reporter assay system (RG088S, Beyotime) according to the manufacturer’s instructions.

### 2.12 miRNA target prediction

MiRNA target genes were predicted using the TargetScan (http://www.targetscan.org/vert_71/), Tarbase (http://microrna.gr/tarbase/), miRDB (http://mirdb.org) and miRanda (http://www.microrna.org/microrna/home.do) databases. We used the databases of RNAhybrid (bibiserv.techfak.uni-bielefeld.de/rnahybrid) to analyze the miRNA-mRNA interaction.

### 2.13 Statistical analysis

Statistical analysis was conducted using GraphPad Prism 10.1 software (GraphPad Software, San Diego, CA). Student’s t-test was employed for comparisons between two groups, while one-way ANOVA was utilized for comparisons among multiple groups. P-values of less than 0.05 were considered statistically significant.

## 3 Results

### 3.1 Establishment of a cellular model of psoriasis, isolation, and characterization of HaCaTs-derived exosomes

**FIGURE 1:**
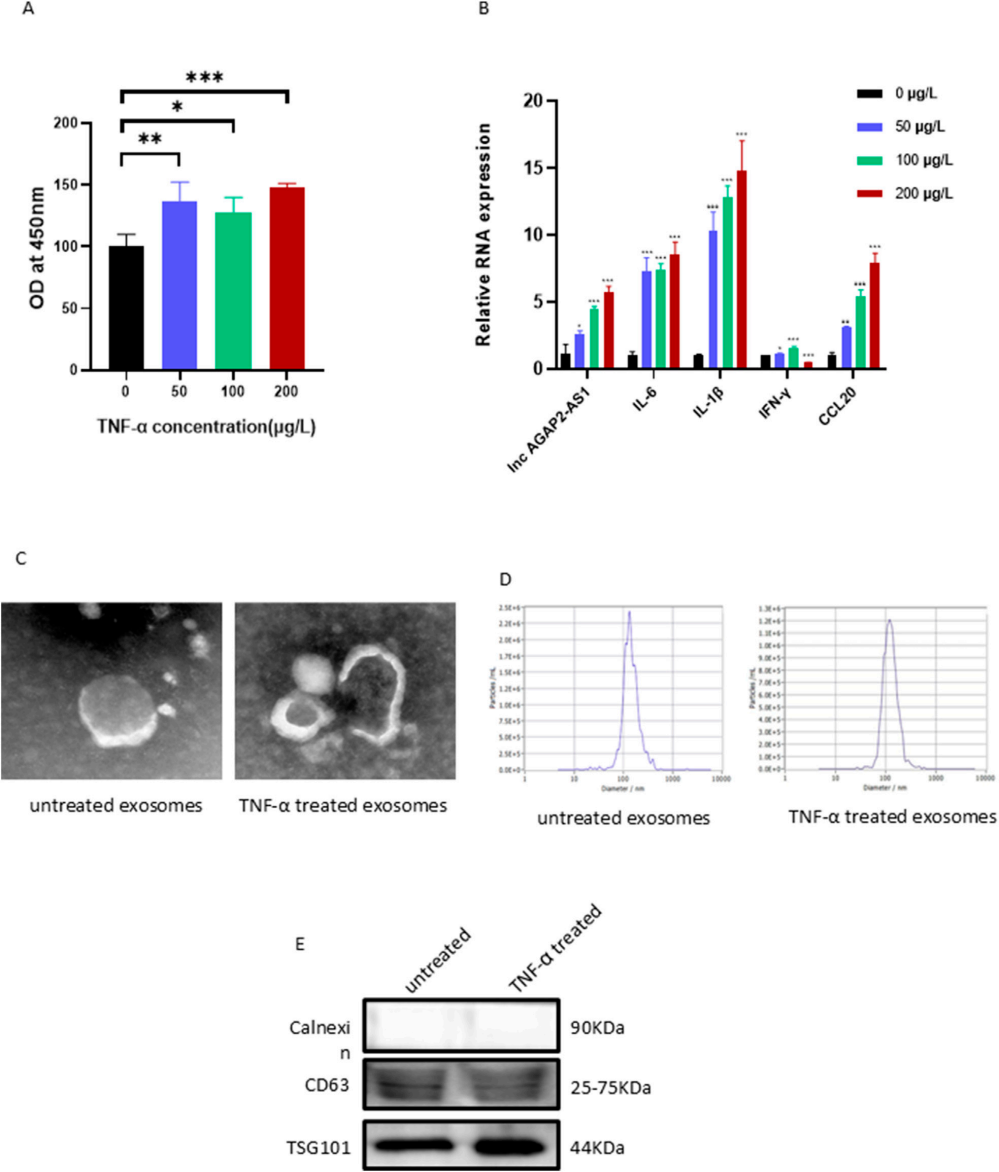
Stimulation of HaCaT cells with different concentrations of TNF-α, isolation and characterization of HaCaT-derived exosomes. **(A)** The viability of the HaCaT cells was increased. **(B)** The mRNA levels of inflammatory factors and chemokines in HaCaT cells. Exosome morphology was assessed by TEM **(C)**, particle size by NTA **(D)**, and marker proteins by Western blot **(E)**. Experiments were repeated three times. *P < 0.05, **P < 0.01, ***P < 0.001.

To establish the psoriatic cell model, HaCaT cells were stimulated with an exosome-free DMEM medium containing different concentrations of TNF-α at cell densities of 70%–80%, and the cell viability and expression levels of various inflammatory factors were examined. As measured by the CCK-8 assay, we found that the viability of the HaCaT cells was significantly increased in response to the stimulation of TNF-α at different concentrations (Figure 1A). Compared to the blank control group, the mRNA expression of AGAP2-AS1, inflammatory cytokines such as IL-6, IL-1β, IFN-γ, and chemokine Ccl20 in HaCaT cells of the experimental group showed a significant concentration-dependent upregulation. Based on the dose-effect relationship and experimental cost-benefit analysis, we ultimately selected 100 μg/L of TNF-α as the stimulus concentration for downstream experiments to simulate the psoriatic microenvironment.

To determine the function of HaCaTs-derived exosomes, we cultured HaCaT cells in an exosome-free medium with or without TNF-α for 24 h to mimic the psoriatic or healthy microenvironment and used an Exosome Extraction and Purification Kit to purify the exosomes. The characteristics of the exosomes were analyzed by various experimental methods such as TEM, NTA, and Western Blot. The experiments revealed that the purified exosomes displayed a typical double membrane structure and spherical form (Figure 1C). The average size of the purified vesicles was 120.6 nm (HaCaTs with TNF-α treatment) and 135.8 nm (HaCaTs without TNF-α treatment), respectively (Figure 1D). In addition, the typical exosomal markers CD63 and TSG101 were found in these extracted and purified exosomes. Calnexin was absent, thus excluding the possibility of endoplasmic reticulum contamination (Figure 1E). Taken together, these data indicate that the isolation and purification of exosomes were successful.

### 3.2 Exosomes from TNF-α-treated HaCaTs promote differentiation of Th1 and Th17 cells

**FIGURE 2:**
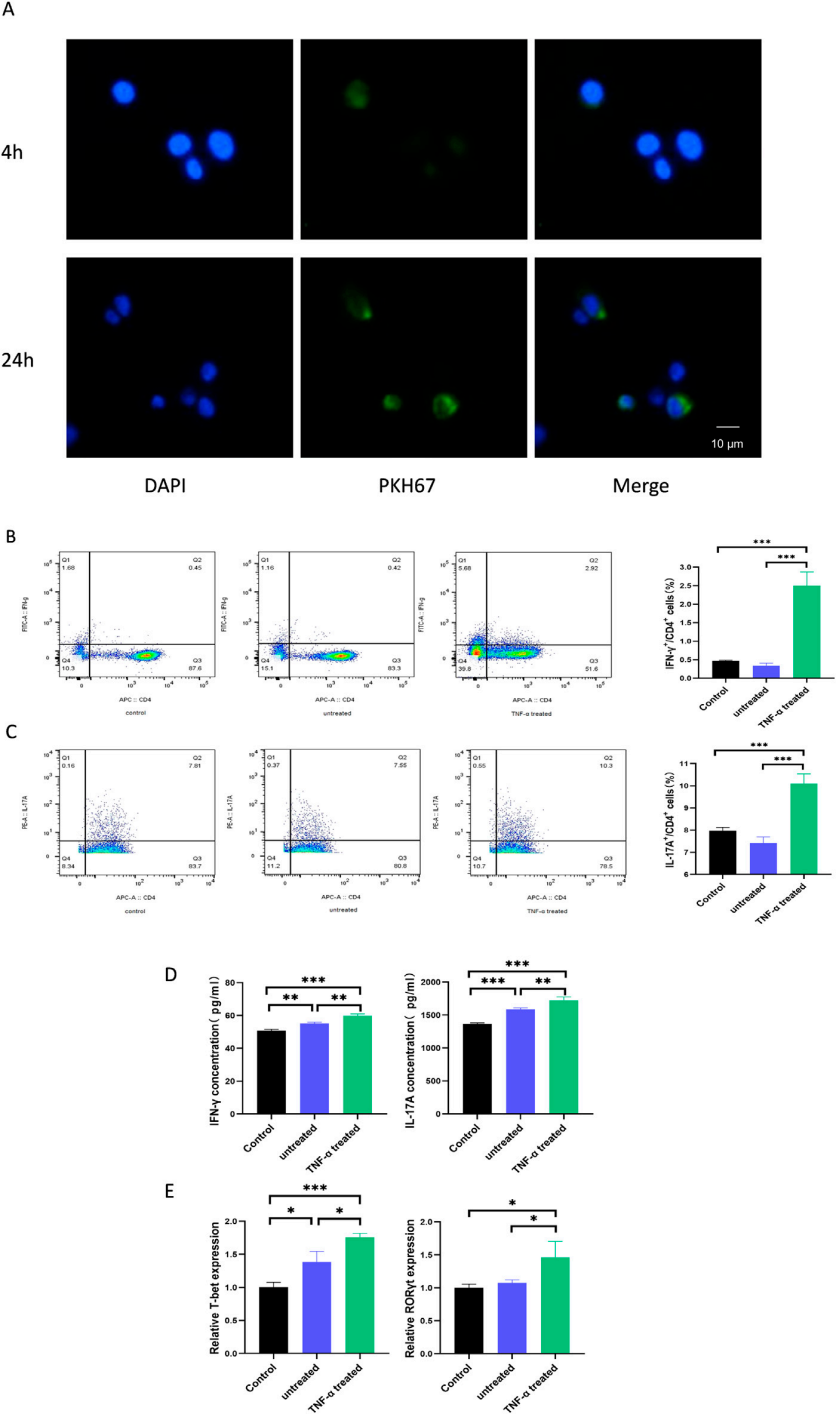
TNF-α-treated HaCaTs-derived exosomes promote the differentiation of Th1 and Th17 cells. **(A)** Representative immunofluorescence images of exosome-treated CD4^+^ T cells. Cell nuclei were counterstained with DAPI (blue), and exosomes were stained with PKH67 (green). After treatment of CD4^+^ T cells with exosomes, the ratio of Th1 **(B)** and Th17 **(C)** cells was detected by flow cytometry; protein levels of relevant cytokines were detected by ELISA **(D)**; mRNA levels of relevant transcription factors were detected by qPCR **(E)**. Th, T helper. Experiments were repeated three times. *P < 0.05, **P < 0.01, ***P < 0.001. ns: no significance.

To determine whether exosomes are taken up by CD4^+^ T cells, the pre-activated CD4^+^ T cells were treated with PKH67-labelled exosomes and monitored by fluorescence microscope. It was found that after 4 h of co-culture, CD4^+^ T cells were able to show clear fluorescence, and the fluorescence intensity inside the cells gradually increased over time. The labeled exosomes were localized in the cytoplasm and around the nucleus of the recipient cells (Figure 2A). This indicates that exosomes are capable of being taken up by CD4^+^ T cells.

To investigate whether HaCaTs can influence infiltrating immune cells in the psoriatic disease state, we isolated CD4^+^ T cells from the peripheral blood of healthy individuals. After 5 days of co-culture with exosomes from the treated and untreated groups, the ratio of Th1 and Th17 cells was determined by flow cytometry. Meanwhile, the expression levels of T-bet and RORγt and the secreted levels of IFN-γ and IL-17A, which are specific for Th1 and Th17, respectively, were assessed by qPCR and ELISA. Interestingly, we found that the percentage of CD4^+^IFN-γ^+^ T cells and CD4^+^IL-17A^+^ T cells was significantly increased by adding exosomes from TNF-α-treated HaCaTs compared to those from untreated HaCaTs (Figures 2B, C). In addition, the protein levels of the cytokines showed similar changes (Figure 2D), and the mRNA levels of T-bet and RORγt were significantly upregulated in CD4^+^ T cells (Figure 2E). These data suggest that TNF-α-treated HaCaTs-derived exosomes promote Th1 and Th17 differentiation in the psoriatic microenvironment.

### 3.3 Exosomes-containing AGAP2-AS1 is responsible for exosome-induced differentiation of Th1 and Th17 cells

In previous studies, we have found differences in the expression of AGAP2-AS1 in healthy individuals and lesional skin tissue from psoriatic patients (Xian et al., 2022). Given the important role of immune cells in psoriasis, we hypothesized that AGAP2-AS1 in HaCaTs could be taken up by CD4^+^ T cells via exosomes, thus promoting the differentiation of Th1 and Th17 cells.

**FIGURE 3:**
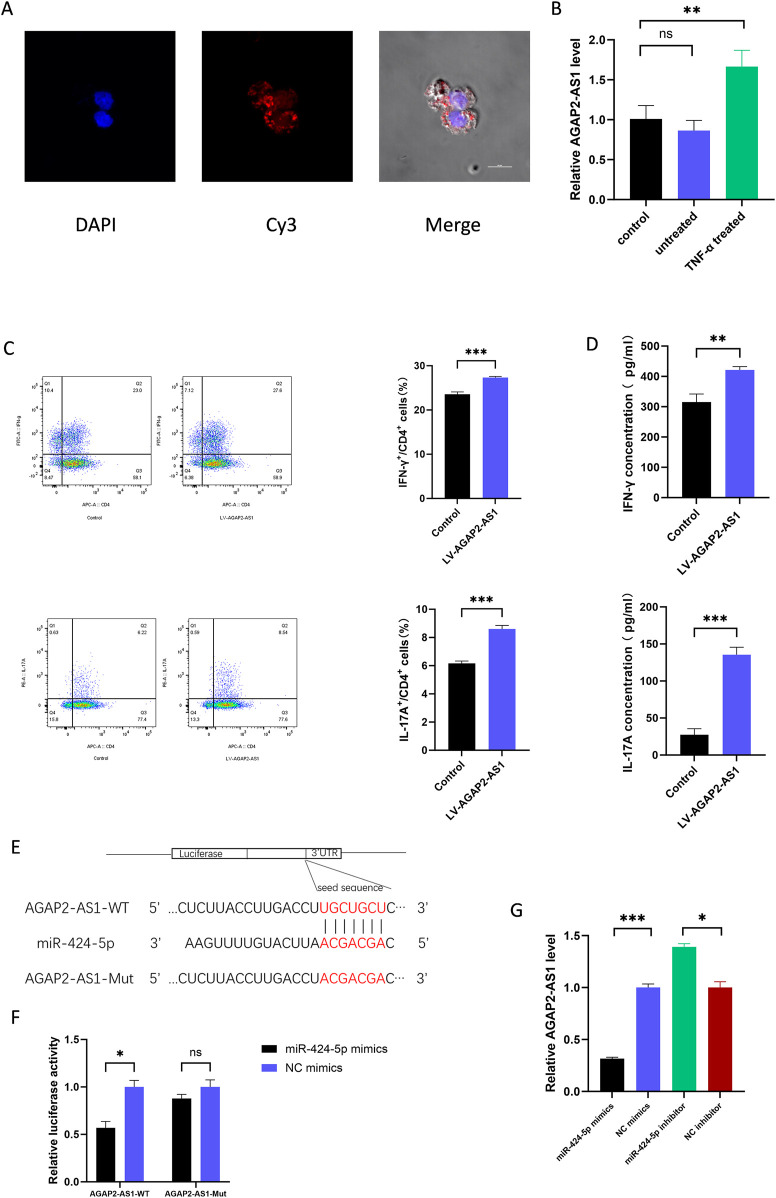
ASAP2-AS1 promotes the differentiation of Th1 and Th17 in CD4^+^ T cells and serves as a sponge for miR-424-5p. **(A)** Fluorescence microscopy was used to detect the subcellular localization of AGAP2-AS1 in CD4^+^ T cells. AGAP2-AS1 was labeled with Cy3, cell nuclei were counterstained with DAPI (blue). **(B)** The expression of AGAP2-AS1 in CD4^+^ T cells after co-incubation of TNF-α-treated HaCaTs-derived exosomes. **(C)** CD4^+^ T cells were transfected with LV-AGAP2-AS1 or LV-NC. The percentage of Th1 and Th17 cells was detected by flow cytometry. **(D)** The protein levels of IFN-γ and IL-17A are measured by ELISA. **(E)** Prediction of the binding sequence between AGAP2-AS1 and miR-424-5p and construction of the AGAP2-AS1 MUT sequence. **(F)** Dual-luciferase reporter gene assay confirmed the binding relationship between AGAP2-AS1 and miR-424-5p in HEK-293T cells. **(G)** AGAP2-AS1 expression level in CD4^+^ T cells transfected with miR-424-5p mimics or NC mimics and miR-424-5p inhibitor or NC inhibitor. WT wild type, MUT mutant type. Th, T helper. Experiments were repeated three times. *P < 0.05, **P < 0.01, ***P < 0.001. ns: no significance.

We labelled AGAP2-AS1 with Cy3 and analysed the cells using laser scanning confocal microscopy. We observed that the labelled AGAP2-AS1 in HaCaTs-exo was taken up by CD4^+^ T cells (Figure 3A). Meanwhile, the expression of AGAP2-AS1 increased after co-incubation of exosomes from TNF-α-treated HaCaTs with CD4^+^ T cells (Figure 3B). Furthermore, overexpression of AGAP2-AS1 on CD4^+^ T cells significantly increased the frequency of CD4^+^IFN-γ^+^ T cells and CD4^+^IL-17A^+^ T cells compared to control cells. ELISA analysis showed similar changes in cytokine protein levels (Figures 3C, D). Taken together, these data indicate that AGAP2-AS1 promotes the differentiation of CD4^+^ T cells to Th1 and Th17 cells via exosomes.

### 3.4 AGAP2-AS1 serves as a sponge for miR-424-5p

In previous studies, we found that AGAP2-AS1 serves as a sponge for miR-424-5p in HaCaTs (Xian et al., 2022), and we suspect that a similar mechanism exists in CD4^+^ T cells. Using Targetscan, we predicted the complementary sequence of AGAP2-AS1 and miR-424-5p to construct mutations (Figure 3E). Dual-luciferase reporter assays were performed to confirm the prediction. The results showed that transfection of miR-424-5p mimics significantly decreased the luciferase activity of 293T cells transfected with wild-type AGAP2-AS1 vector, but did not affect the luciferase activity of transfected with mutant AGAP2-AS1 vector (Figure 3F). In addition, upregulation of miR-424-5p significantly decreased the expression of AGAP2-AS1, whereas downregulation of miR-424-5p increased the expression of AGAP2-AS1 (Figure 3G). Thus, AGAP2-AS1 acted as a miR-424-5p sponge in CD4^+^ T cells.

### 3.5 SGK1 is the target gene of miR-424-5p in CD4^+^ T cells

**FIGURE 4:**
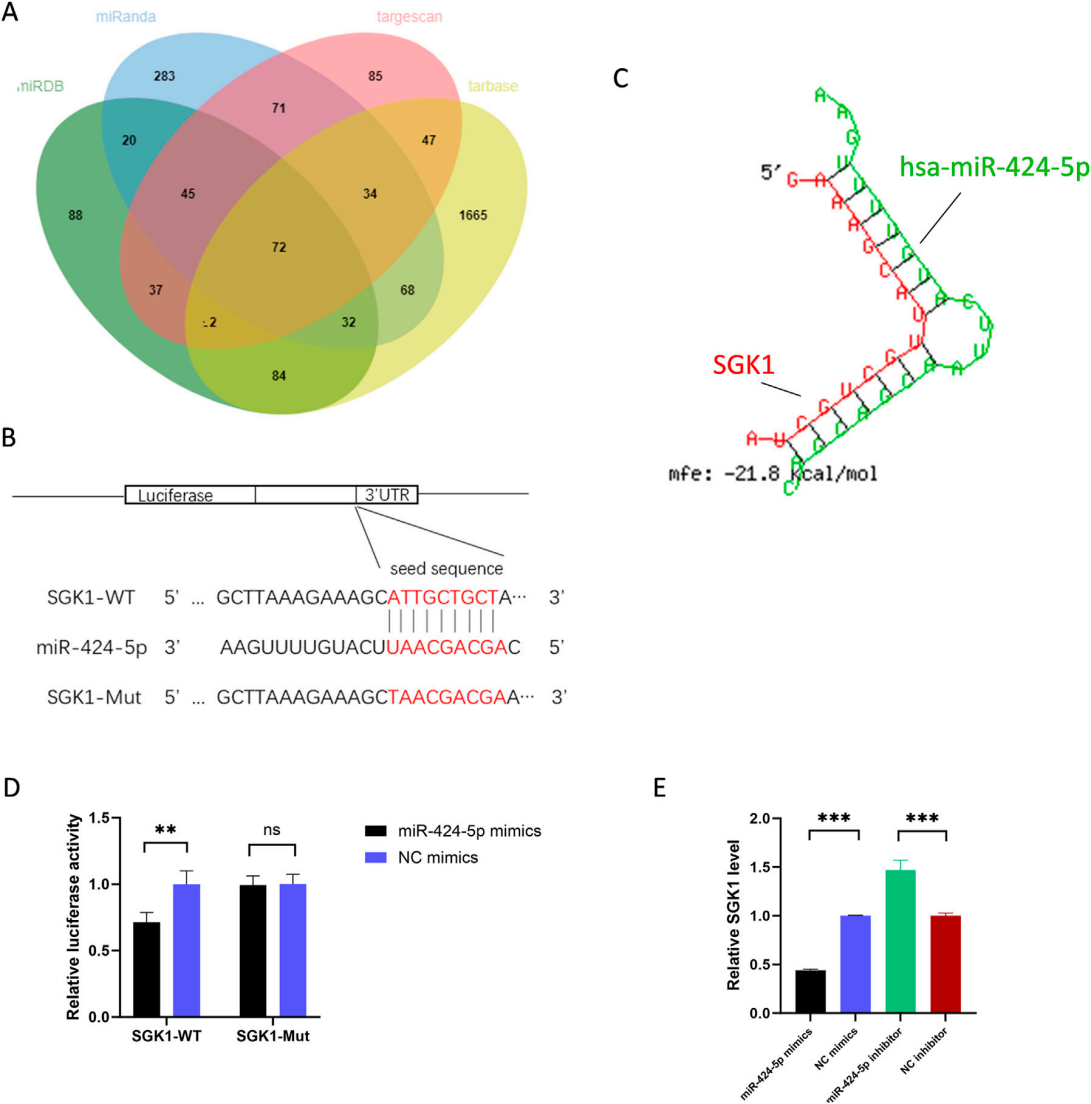
SGK1 is the target gene of miR-424-5p. **(A)** Target genes of miR-424-5p predicted by TargetScan, Tarbase, miRDB, and miRanda. **(B)** Prediction of the binding sequence between SGK1 and miR-424-5p and construction of the SGK1 MUT sequence. **(C)** Prediction of SGK1 3′-UTR binding sites for miR-424-5p using RNA hybrid tools. **(D)** Dual-luciferase reporter gene assay confirmed the binding relationship between SGK1 and miR-424-5p in HEK-293T cells. **(E)** SGK1 expression level in CD4^+^ T cells transfected with miR-424-5p mimics or NC mimics and miR-424-5p inhibitor or NC inhibitor. WT wild type, MUT mutant type. Experiments were repeated three times. *P < 0.05, **P < 0.01, ***P < 0.001. ns: no significance.

We used TargetScan, Tarbase, miRDB, and miRanda, databases to predict the common target genes of miR-424-5p and display them in Venn diagrams. Of the 104 candidate target genes, Serum and glucocorticoid-regulated kinase 1 (SGK1) was selected for further mechanistic exploration due to its key role in immune regulation (Figure 4A). RNAhybrid predicted a strong combination ability of miR-424-5p with SGK1 based on the minimum free energy (mfe) (Figure 4C). SGK1 is a serine/threonine kinase belonging to the protein kinase A, G, and C (AGC) family (Jang et al., 2022). SGK1 regulates essential cellular processes such as proliferation, survival, and apoptosis. SGK1 has been reported to play a key role in the development of Th17 cells (Wu et al., 2018).

We constructed SGK1-WT and SGK1-MUT plasmids (Figure 4B) and co-transfected them with miR-424-5p mimics or NC mimics into HEK-293T cells. The results showed that compared with the control group, the luciferase activity was significantly reduced in cells co-transfected with SGK1-WT and miR-424-5p mimics. However, there was no significant difference in luciferase activity between cells co-transfected with SGK1-3′UTR-MUT and miR-424-5p mimics and the control group (Figure 4D). In addition, upregulation of miR-424-5p significantly reduces the SGK1 expression, while downregulation of miR-424-5p has the opposite effect (Figure 4E). Overall, SGK1 is the target gene of miR-424-5p in CD4^+^ T cells.

### 3.6 AGAP2-AS1 promotes CD4^+^ T cell differentiation through the miR-424-5p/SGK1 axis

**FIGURE 5:**
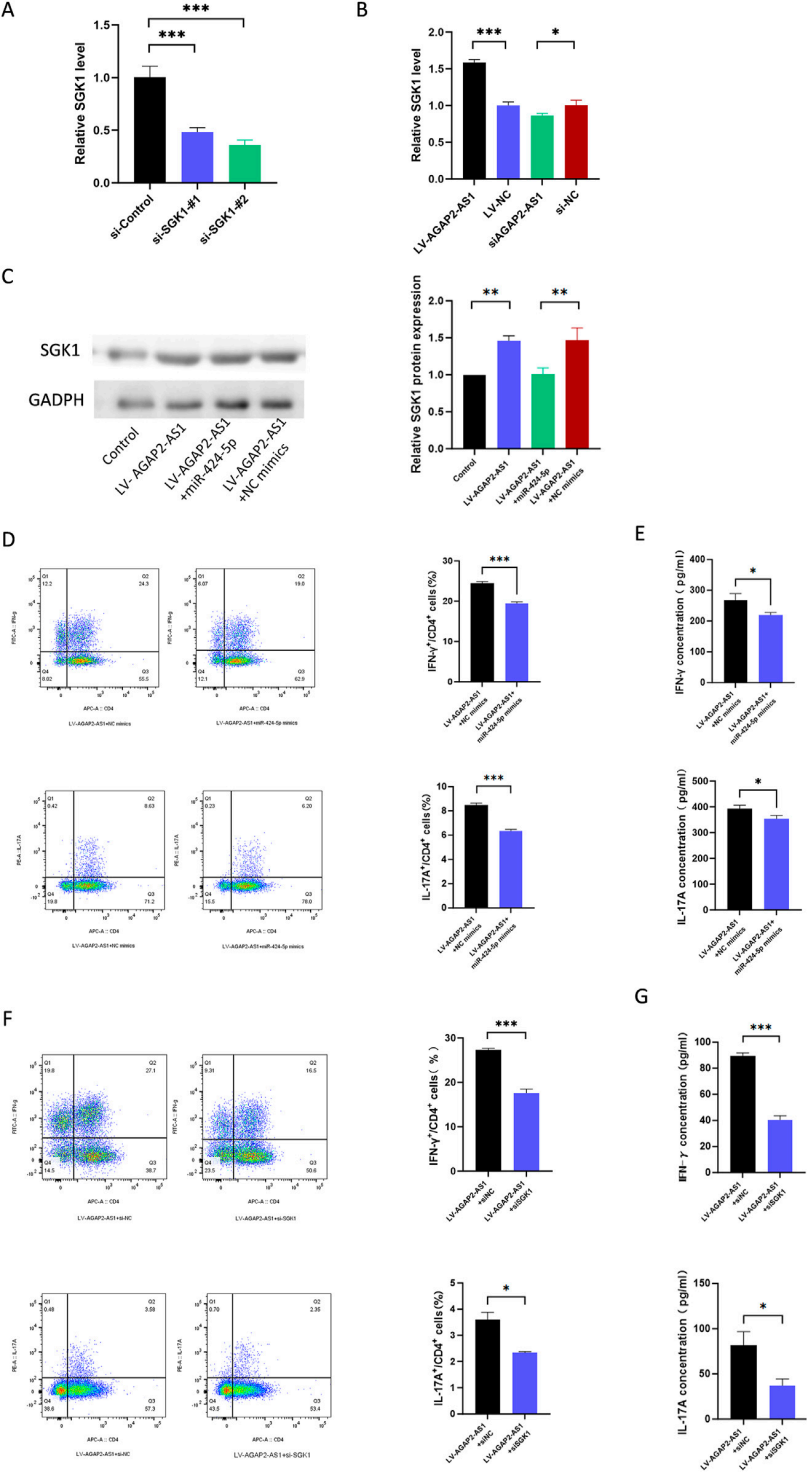
AGAP2-AS1/miR-424-5p regulated CD4^+^ T cell differentiation via the SGK1 signaling pathway. **(A)** The relative expression levels of SGK1 in 293T cells that were transfected with siRNA targeting SGK1. **(B)** SGK1 expression level in CD4^+^ T cells transfected with LV-AGAP2-AS1 or LV-NC and si-AGAP2-AS1 or si-NC. **(C)** Western blot confirmed that transfection of AGAP2-AS1 in CD4^+^ T cells promoted the expression of SGK1, and the miR-424-5p mimics reversed this process. **(D)** Flow cytometry analysis of the percentages of Th1 and Th17 cells in CD4^+^ T cells transfected with LV-AGAP2-AS1 and miR-424-5p mimics or NC mimics. **(E)** The protein levels of IFN-γ and IL-17A are measured by ELISA. **(F)** Flow cytometry analysis of the percentages of Th1 and Th17 cells in CD4^+^ T cells transfected with LV-AGAP2-AS1 and SGK1 siRNA or NC siRNA. **(G)** The protein levels of IFN-γ and IL-17A are measured by ELISA. The experiments were repeated three times. *P < 0.05, **P < 0.01, ***P < 0.001.

We conducted a series of rescue experiments in CD4^+^ T cells. First, we constructed siRNA targeting SGK1 and confirmed the knockdown efficiency of SGK1 (Figure 5A). Our data showed that the upregulation of AGAP2-AS1 increased the expression of SGK1, while the downregulation of AGAP2-AS1 was the opposite (Figure 5B). Meanwhile, miR-424-5p mimics could reverse the upregulation of SGK1 induced by AGAP2-AS1 (Figure 5C).

To evaluate whether AGAP2-AS1 plays a role in CD4^+^ T cell differentiation through the miR-424-5p/SGK1 axis, we performed corresponding rescue experiments. Flow cytometry showed that miR-424-5p mimics could partially reverse AGAP2-AS1-induced CD4^+^ T cell differentiation, and ELISA experiments confirmed the same results (Figures 5D, E). Meanwhile, siSGK1 could reverse AGAP2-AS1-induced CD4^+^ T cell differentiation (Figures 5F, G). Overall, these results validate that AGAP2-AS1 promotes CD4^+^ T cell differentiation through the miR-424-5p/SGK1 axis.

## 4 Discussion

Psoriasis is a chronic, relapsing, immune-mediated inflammatory skin disease characterized by excessive proliferation and abnormal differentiation of epidermal cells. Immune abnormalities, particularly CD4^+^ T lymphocyte differentiation and dysfunction, are at the core of its pathogenesis. Several key cytokines, including TNF-α, IL-12/IL-23, and IL-17A, play a crucial role in the pathogenesis and development of psoriasis. Among these, the IL-23/Th17 axis represents the core inflammatory pathway in psoriasis, with IL-17 acting as a downstream effector factor of the primary inflammatory cascade. Currently, biologics targeting IL-17, such as secukinumab and ixekizumab, have shown promising results in clinical trials for treating moderate to severe plaque psoriasis. In addition, there have been advances in the clinical use of agents that modulate the upstream IL-23 or downstream molecular pathways of IL-17. These advances highlight the importance of IL-17 and emphasize the critical importance of studying CD4^+^ T cell differentiation and secretion of inflammatory factors in elucidating the pathogenesis of psoriasis (Conrad and Gilliet, 2018). Our previous research identified elevated levels of AGAP2-AS1 in psoriatic lesions and demonstrated its role in promoting keratinocyte proliferation through miR-424-5p. However, the associated immune mechanisms remained unexplored until now.

Exosomes, phospholipid bilayer structures derived from the cell membrane, facilitate the transfer of biological information, including various RNAs, lipids, and proteins between cells. They play an important role in inflammatory skin diseases (Jiang et al., 2019; Shao et al., 2020). Previous studies have reported that exosomes derived from cytokine-treated keratinocytes can affect the differentiation of Th1 and Th17 (Jiang et al., 2021). AGAP2-AS1, a long non-coding RNA, has been widely confirmed as an oncogenic lncRNA in various cancers, including lung, breast, and colon cancer (Ma et al., 2024). Additionally, it has been observed that AGAP2-AS1 is dysregulated in the skin tissue of patients with systemic sclerosis and may be involved in the pathogenesis of this disease (Messemaker et al., 2025). In addition, reports indicate that the glioma-derived extracellular vesicle AGAP2-AS1 promotes glioma proliferation and metastasis by mediating myeloid-derived inhibition of cell secretion of TGF-β1 (Tian, 2024). We observed that TNF-α-treated HaCaTs-derived exosomes can accelerate the psoriasis process by delivering AGAP2-AS1 to promote CD4^+^ T cell differentiation towards Th1 and Th17 cells and secretion of more inflammatory factors such as IFN-γ and IL-17A, which affect the immune microenvironment.

MicroRNAs (miRNAs) are endogenous small RNAs, approximately 20–24 nucleotides in length, that play a variety of important regulatory roles in cells. Using the microRNA target database, we identified that AGAP2-AS1 contains a binding site for miR-424-5p. MiR-424-5p has been reported to be involved in the pathogenesis of various cancers (Xuan et al., 2022). Additionally, the downregulation of miR-424-5p has been associated with overgrowth and excessive proliferation of psoriatic keratinocytes (Jinnin, 2015). Elevated expression of miR-424-5p has also been found in PBMCs of patients with autoimmune disease pemphigus, suggesting its potential role in pathogenesis (Wang et al., 2017). In this study, we elucidated the relationship between AGAP2-AS1 and miR-424-5p. Overexpression of miR-424-5p can inhibit the expression of AGAP2-AS1, whereas downregulation of miR-424-5p can promote the expression of AGAP2-AS1. We confirmed that AGAP2-AS1 acts as a sponge for miR-424-5p in CD4^+^ T cells.

Here, we found that SGK1 is a functional target of miR-381-3p, mediating the regulatory effect on CD4^+^ T cell differentiation. Dysregulation of SGK1 expression can lead to various diseases, including hypertension, cancer, and autoimmune, and neurodegenerative diseases (Bian et al., 2023; Jang et al., 2022). SGK1 has been shown to regulate Th17 differentiation via IL-23 and is one of the top genes in the total gene transcript of Th17 differentiation *in vitro* (Wu et al., 2013). Our data reveal that the expression of miR-424-5p has a negative regulatory effect on the expression of SGK1. The rescue experiment results showed that AGAP2-AS1 upregulated SGK1 through miR-424-5p, which further promoted the differentiation of Th1 and Th17 cells. Furthermore, our rescue experiments have also observed that overexpressing miR-424-5p or si-SGK1 exerts a certain inhibitory effect on CD4^−^T subsets. This suggests that miR-424-5p and SGK1 may play significant roles in other immune-related diseases as well.

In conclusion, our study shows that psoriatic HaCaTs deliver AGAP2-AS1 to CD4^+^ T cells via exosomes and promote Th1 and Th17 differentiation via the AGAP2-AS1/miR-424-5p/SGK1 axis. This indicates that HaCaTs can transmit information to CD4^+^ T cells through exosomes and that AGAP2-AS1 plays an important role in this process. This provides us with a new approach to achieve plasticity in CD4^+^ T cell differentiation by regulating the content of lncRNA, thereby improving the progression of psoriasis. However, verifying this mechanism in specific cell subpopulations poses certain difficulties in animal experiments. Our study innovatively adopted a primary human CD4^+^ T-cell model, which can truly reflect human T-cell differentiation mechanisms, thus demonstrating a certain degree of translational applicability.

## Data Availability

The original contributions presented in the study are included in the article, further inquiries can be directed to the corresponding author.
